# Management and risk factors for maternal morbidity of eclampsia in a Moroccan teaching hospital

**DOI:** 10.1186/cc9934

**Published:** 2011-03-11

**Authors:** Y Zarrouki, M Boutbaoucht, Y El Waggagui, G El Adib, S Younous

**Affiliations:** 1 Mohammed VI Teaching Hospital, Marrakesh, Morocco

## Introduction

Eclampsia is a serious complication of pregnancy, it remains a frequent condition in our context. The aim of this study is to measure the incidence of eclampsia, its risk factors associated with adverse maternal outcome and to identify its most common presentations in our practice.

## Methods

Through a prospective descriptive study spread over 1 year (November 2009 to October 2010), all cases of eclampsia gathered in the maternity ICU of Marrakesh Teaching hospital are included, and epidemiological and prognostic data were analyzed by either chi-squared analysis or the unpaired Student test as appropriate.

## Results

The incidence of eclampsia was 6.68/1,000 deliveries, it is behind 11% of hospitalizations in our ICU (59 cases during study period) with 87% of patients referred from all southern Morocco. Sixty-two percent of seizures occurred antepartum, 20% during labor and 18% postpartum. Two peaks of age are observed, 22 ± 5 years and 36 ± 4 years. Major maternal complications included HELLP syndrome (12%), abruptio placentae (8%), disseminated intravascular coagulopathy (8%), pulmonary edema (5%), acute renal failure requiring dialysis (4%), aspiration pneumonia (3%) and neurologic complications (3%) including hemorrhage, ischemia and cerebral venous thrombosis. Maternal mortality was 6.7% and perinatal mortality was 16.9%. Parturients with antepartum eclampsia have significantly higher incidences of HELLP syndrome (14% vs. 6%; *P *= 0.02) and abruptio placentae (12% vs. 4%; *P *= 0.006) than did those in whom eclampsia developed intrapartum and postpartum. In contrast, women with postpartum eclampsia were more unlikely to have acute renal failure (7% vs. 2%; *P *= 0.005) and neurologic complications (5% vs. 1%; *P *= 0.001) than were those with antepartum eclampsia. In addition, older women develop more renal failure than younger ones (9% vs. 2%; *P *= 0.001).

## Conclusions

Pregnancies complicated by eclampsia are purveyors of high maternal morbidity and mortality. Antepartum and postpartum cases were more severe than intrapartum cases; the same observation is made among older women.

